# COVID-19 and Liver Damage: Narrative Review and Proposed Clinical Protocol for Critically ill Pediatric Patients

**DOI:** 10.6061/clinics/2020/e2250

**Published:** 2020-11-02

**Authors:** Michele Luglio, Uenis Tannuri, Werther Brunow de Carvalho, Karina Lucio de Medeiros Bastos, Isadora Souza Rodriguez, Cintia Johnston, Artur Figueiredo Delgado

**Affiliations:** I Centro de Terapia Intensiva do Instituto da Crianca e do Adolescente do Hospital das Clínicas da FMUSP, Sao Paulo, SP, BR; II Cirurgia Pediatrica e Transplante Hepatico do Instituto da Crianca e do Adolescente do Hospital das Clínicas da FMUSP, Sao Paulo, SP, BR; III Hepatologia Pediatrica do Instituto da Crianca e do Adolescente do Hospital das Clínicas da FMUSP, Sao Paulo, SP, BR; IV Unidade de Apoio Anestesico do Instituto Central do Hospital das Clínicas da FMUSP, Sao Paulo, SP, BR

**Keywords:** Coronavirus, COVID-19, Liver, Pediatric

## Abstract

SARS-CoV-2 shares nearly 80% of its’ genomic sequence with SARS-CoV and MERS-CoV, both viruses known to cause respiratory symptoms and liver impairment. The emergence of pediatric cases of multisystem inflammatory syndrome related to the SARS-CoV-2 infection (PIM-TS) has raised concerns over the issue of hepatic damage and liver enzyme elevation in the critically ill pediatric population with COVID-19. Some retrospective cohorts and case series have shown various degrees of ALT/AST elevation in SARS-CoV-2 infections. A limited number of liver histopathological studies are available that show focal hepatic periportal necrosis. This liver damage was associated with higher levels of inflammatory markers, C-reactive protein (CRP), and pro-calcitonin. Proposed pathophysiological mechanisms include an uncontrolled exacerbated inflammatory response, drug-induced liver injury, direct viral infection and damage to cholangiocytes, hypoxic-ischemic lesions, and micro-thrombosis in the liver. Based on the physiopathological characteristics described, our group proposes a clinical protocol for the surveillance, evaluation, management, and follow-up of critically ill pediatric COVID-19 patients with liver damage.

## INTRODUCTION

Since December 2019, an emergent primarily respiratory illness caused by a new strain of coronavirus (Severe Acute Respiratory Syndrome Coronavirus 2 - SARS-CoV-2), later named COVID-19 (coronavirus disease 2019), has resulted in significant numbers of critical care admissions worldwide.

SARS-CoV-2 shares nearly 80% of its’ genomic sequence with SARS-CoV and 50% with Middle East respiratory syndrome coronavirus (MERS-CoV), both of which are known to cause severe respiratory symptoms and liver impairment (60% of patients with SARS-CoV and some reports in patients with MERS-CoV) (1). Recent studies have documented liver function abnormalities, represented by alanine aminotransferase (ALT) or aspartate aminotransferase (AST) in 14-53% of COVID-19 adult patients (2).

Between March and May 2020, the emergence of pediatric cases of multisystem inflammatory syndrome related to SARS-CoV-2 infection (PIM-TS) has been suggested, and data from 58 patients were recently described (3). Among these patients, stratified in different clinical categories, median (IQR) values of ALT ranged from 26 (12-141) to 86 (34-129), raising concerns over the important question of hepatic involvement in critically ill pediatric COVID-19 patients (3).

Taking this into account, our group performed a brief literature review in order to summarize the available evidence on the topic of liver compromise in the context of SARS-CoV-2 infection, its’ particularities, and possible physiopathological mechanisms. With these concepts in mind, we developed a protocol for the assessment and care of pediatric patients with COVID-19 and liver function abnormalities.

### Liver Tests Abnormalities in COVID-19 Infections

With the development of a greater body of evidence for the clinical features of COVID-19, some case studies have shown that SARS-CoV-2 infected patients may present with varying degrees of hepatic enzyme (AST/ALT) elevation. Zhang et al., in a recently published Comment in The Lancet, compiled data from seven different studies (2,4-10). These adult studies showed that liver dysfunction, represented by AST elevations could occur in SARS-CoV-2 infections, even in the absence of previous hepatic disease (reported in 2-11% of the patients in the studies mentioned).

Shi et al. (8), in a descriptive study, demonstrated that such transaminase elevations were more common in patients in the symptomatic phase than in patients in the subclinical phase of COVID-19 (confirmed by thoracic computed tomography (CT) scans). These data are consistent with the observation that liver injury and dysfunction are more prevalent in severe and critical cases of COVID-19.

Cai et al. (11), recently proposed a classification for the various patterns of liver test abnormalities found in COVID-19 infections. The study defined three patterns: hepatocellular, cholestatic, and mixed. These were based on the values of ALT/AST, gamma-glutamyl transferase (GGT), alkaline phosphatase (ALP), and total bilirubin. A total of 417 patients with COVID-19 and at least one liver function test abnormality was included, with 90 patients (21.5%) classified as having liver injury (ALT/AST>3x normal upper limit and/or GGT/ALP>2x upper limit). The presence of liver test abnormalities on hospital admission increased the risk of severe pneumonia in this population, especially among those with the hepatocellular or mixed type. This condition indicated a higher mortality rate. Patients experienced a substantial increase in the incidence of hepatic injury during hospital stays, even with a low prevalence of previous liver disease. This may suggest a direct viral infection of liver cells or adverse effects of drugs used during hospitalization. Liver biopsy specimens from a patient who died from COVID-19 in this cohort (11) raised the possibility that altered liver enzymes could be caused by drugs used in the treatment of sepsis, or shock.

A cohort of COVID-19 patients admitted to a Shangai Hospital (12) showed that 37.2% of the included patients had abnormal liver function tests on admission. ALP elevations were the less common liver enzyme alterations, suggesting that even with a high expression of angiotensin-converting enzyme 2 (ACE-2) in the bile ducts, direct damage to these structures by the virus may not occur. The levels of ALT/AST elevation detected in the study were mild and more commonly associated with higher inflammatory indices, such as elevated C-reactive protein (CRP) and procalcitonin (PCT). Although this may suggest a role for uncontrolled inflammation in the pathogenesis of COVID-19 related liver injury, this observation cannot justify the presence of abnormal liver function tests found in patients with mild SARS-CoV-2 infection.

Liver test abnormalities can be found in patients with COVID-19. This finding is associated with a longer hospital stay and a more severe clinical course. The exact physiopathological mechanisms are not completely understood, with potential roles for direct viral lesions in hepatic/cholangiocytes cells, inflammatory damage, hypoxic/shock-related circulatory compromise, endothelial dysfunction, microthrombi formation, and drug toxicity all under consideration.

In Table 1 below, we present different patterns of liver test alterations related to COVID-19 proposed pathogenesis for hepatic damage:

### Liver Biopsy and Necropsy Findings

A limited number of pathological studies are available to date concerning the livers of COVID-19 patients. Histopathologic changes include focal hepatic necrosis adjacent to the terminal hepatic vein and focal periportal hepatic necrosis (14). Steatosis was reported in one case (15), while multifocal hepatic necrosis was significant even without clear inflammatory cell infiltration and portal tract inflammation (16,17).

As mentioned above, Cai et al. (11) in one liver biopsy from a deceased 69-year-old patient, no obvious portal inflammation was shown. In the preserved structures of the interlobular bile ducts, veins, and arteries, a few hepatocytes with mild vesicular steatosis with watery degeneration and slight inflammatory cell infiltrate in the hepatic sinuses were observed. These findings may be related to drugs used in the treatment of COVID-19 hypoxia and ischemia.

### Potential Pathophysiological Mechanisms for COVID-19 related Hepatic Lesions

Recent findings point out that SARS-CoV-2 enters alveolar epithelial cells by binding with ACE-2 receptors (18,19). ACE-2 receptors are expressed in the liver tissue (19), precisely on cholangiocytes, a finding that suggests a potential direct result of the virus’ damage to the bile duct epithelial cells. Nevertheless, expressive canalicular enzyme elevations or pathological findings of bile duct compromise in COVID-19 patients has not been reported to date.

The presence of viral particles in the feces of infected patients suggests gastrointestinal tract involvement by the virus (20). This is another observation that illustrates the potential direct effect of SARS-CoV-2 on hepatic tissue, given the close relationship between the bowel and the liver (13). The exact mechanisms of this proposed direct damage pathway are yet to be clarified.

Taking into account the above-mentioned findings, it is more probable that the hepatic damage observed in SARS-CoV-2 infections may be secondary, related to hepatotoxic medications, a systemic inflammatory response, severe hypoxia, or multiple organ dysfunction syndrome (DMOS).

Pro-inflammatory cytokines (IL-2R, IL-6, IL-10, TNF-alfa), as well as inflammatory markers (ferritin, PCT, CRP) are markedly elevated in severe cases of COVID-19 (21), leading to an inflammatory &quot;storm&quot;. The virus is also able to induce activation of toll-like receptors and killer T lymphocytes (22), sparking cellular apoptosis and necrosis, release of damage related patterns and stimulation of a vicious cycle of multiple injuries to various organs and cells, the liver included.

In the context of this uncontrolled exacerbated inflammatory process, endothelial cells are central to some pathological aspects of the disease. Alveolar vascular endothelium, activated by inflammation, leads to neutrophil extracellular traps stimulation, an immunological process (23). It triggers microthrombi formation in the lungs, which can further aggravate the pulmonary effects of SARS-CoV-2 (24). This hypercoagulative condition may potentially extend itself to other organs and tissues, affecting the liver and promoting hepatic damage. Autopsies performed in Wuhan patients show signs of micro-thrombosis and hepatic sinusoidal congestion, adding evidence to this observation (25,26).

The initial main concerns regarding SARS-CoV-2 infection in severe acute respiratory syndrome were focused on its’ capacity to induce severe hypoxia that was refractory to the administration of high inspired fractions of oxygen and high mean airway pressures. Animal models (27) show that hypoxia is capable of hepatic cell death and infiltration of inflammatory cells, with lipid accumulation and an increase in oxygen reactive species (28). Hypoxic liver damage is marked by ALT/AST elevations due to oxygen imbalance. This indicates that severe hypoxia may be one of the physiopathological components of the hepatic damage in COVID-19.

Many of the drugs used in the treatment of COVID-19 patients and their complications during hospitalization are considered capable of inducing some extent of liver damage. In a study by Fan et al. (12), a high proportion of patients with abnormal liver function tests had been prescribed lopinavir/ritonavir during their hospital stay. This indicates a potential role for certain medications in the liver damage observed in these patients. This finding is consistent with some of the previously mentioned liver biopsy findings (11,14-17).

Drug-induced liver injury (DILI) is an important differential diagnosis in critically ill patients, mainly due to polypharmacy. Some drugs show a clear and documented link to liver injury, such as paracetamol, chlorpromazine, halothane, isoniazid, and amoxacilin-clavulanate (29). Bjornsson ES, (30) classified drug toxicity in four different categories, based on the LiverTox^®^ (31) website data. Category A is comprised of drugs with more than 50 published reports of association with DILI, excluding herbal and dietary supplements. Category B is comprised of drugs with less than 50 and more than 12 reports on hepatotoxicity. In Table 2 below, the main agents of both groups, with their potential uses in critically ill COVID-19 patients are shown.

The multiplicity of physiopathological processes involved in COVID-19 related liver damage as well as the ubiquitous expression of ACE-2 receptors through the organism, has a potential role in organ crosstalk in the pathogenesis of hepatic damage related to SARS-CoV-2 infection.

Expression of ACE-2 on the luminal gut epithelium makes this organ a direct target of SARS-CoV-2 and can lead to increased permeability, augmented inflammation, and dysregulation of the intestinal microbiome (32). This process can promote gut barrier disruption, that increases the risk of bacterial translocation leading to gram-negative sepsis. The close relationship between the liver and the intestinal tract, given the portal circulation and the biliary tree, the so called “gut-liver axis (33), makes the organ chronically exposed to gut-derived factors and bacteria, a mechanism that can be partially responsible for the observed liver damage in COVID-19 patients.

**Figure 1:**
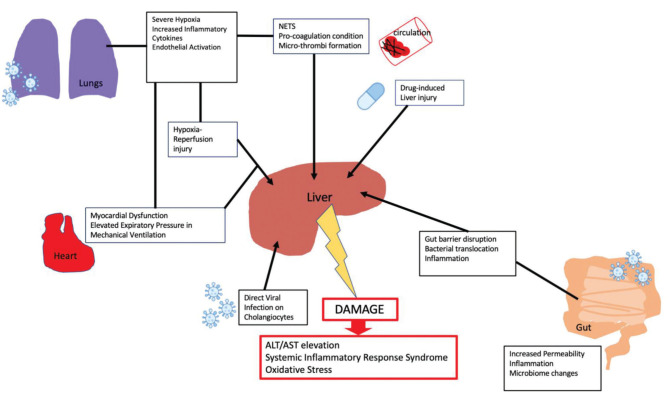
Potential Pathophysiological mechanisms of COVID-19 Liver Damage.

In Figure 1 below, the potential pathophysiological mechanisms of COVID-19 liver damage are summarized.

Although considered rare, pediatric chronic liver disease comprises a group of conditions that aggregates the severity of SARS-CoV-2 infection. The precise mechanisms involved, with careful consideration of the further hepatic function deterioration among these patients requires thorough investigation. The systemic immunocompromised status of cirrhosis can also make these patients more susceptible to COVID-19.

Observations of adult patients with nonalcoholic fatty liver disease (NAFLD) show that high cytokine levels related to chronic liver conditions may lead to rapid progressive hepatic damage in SARS-CoV-2 infections. This results in a disease course that is possibly more severe and further aggravates basal hepatic dysfunction (34).

### Discussion and Proposed Clinical Protocol

Significant variability was found when analyzing ALT/AST elevations in SARS-CoV-2 infections (4-12). GGT and ALP elevations are uncommon in the previously described adult studies (11,12).

Even with the high expression of ACE-2 in cholangiocytes, direct bile duct damage by coronavirus may not be as an important issue, as previously considered. The apparent absence of viral inclusions on liver biopsies as well as the pattern of liver damage, consistent with hypoxic and ischemic injury, adds to this discussion.

The participation of drug hepatotoxicity in COVID-19 induced liver damage has been proposed in previous studies (11,12,16). Drug-induced liver damage may be an important contributing factor to this multifactorial condition.

Analysis of concomitant elevations in lactate dehydrogenase (LDH) and creatinine kinase may be required, especially when considering that many COVID-19 patients present with myalgia or some degree of myositis (34,35). AST elevations found in this context could be attributed, to some extent, to muscular damage (36).

Previous reports on a PIM-TS group of patients (3,37) showed a considerably higher incidence of ALT/AST elevations when compared to the whole pediatric COVID-19 population. This observation may indicate the role of a systemic uncontrolled inflammatory response in the generation of liver damage.

The role of organ crosstalk in the pathogenesis of COVID-19 (32) is now better understood. The close relationship between the liver and the gut, the so called “liver-gut axis” (bacterial translocation and increased inflammation) and the pro-coagulation condition lead by lung endothelial activation can impose intense stress on the liver tissues. This leads to hepatic damage demonstrated by high liver enzymes.

Previous adult studies have shown that the presence of previous hepatic dysfunction or cirrhosis is associated with an increased risk of severe COVID-19 infection (38). Additionally, patients with chronic liver disease may present with worsening liver function tests in SARS-CoV-2 infections (38-40). This is a hypothesis consistent with previous observations of acute decompensation of liver function tests in cirrhotic patients with acute bacterial or viral infections (41). Although considered rare in pediatric patients, careful consideration of these patients must be initiated as a result of the SARS-CoV-2 infection and its’ effects (42).

**Figure 2:**
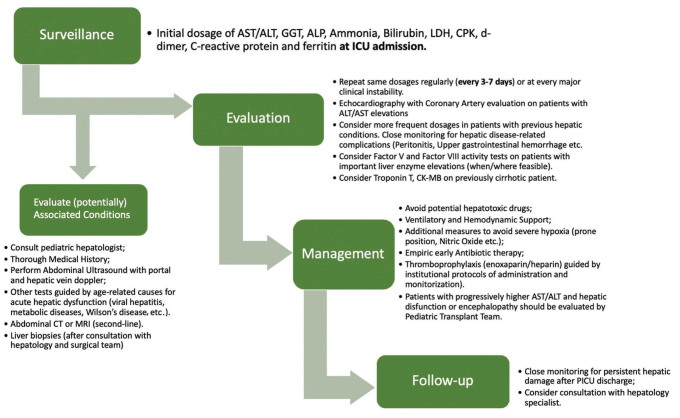
Proposed Surveillance, Evaluation, Management and Follow-up Guidelines for Pediatric Patients with COVID-19 associated liver damage.

Considering the observations above, our group proposes a protocol for the surveillance, evaluation, and management of critical pediatric COVID-19 patients with previous or new liver damage (Figure 2).

The initial evaluation of potential hepatic damage in pediatric COVID-19 shall include AST/ALT, GGT, ALP, Ammonia, Bilirubin, lactate dehydrogenage (LDH), creatinine phosphokinase (CPK), d-dimer, C-reactive protein (CRP) and ferritin evaluations on Intensive Care Unit (ICU) admission. These tests focus on surveillance of hepatocellular and biliary cell damage as well as the evaluation of potential interference with the results by myositis (denoted by elevations on CPK and LDH) and the roles of increased inflammation and pro-thrombotic states in this disease context.

It is necessary to address the potential presence of previously unknown hepatic diseases that may decompensate due to the development of SARS-CoV-2 infection as well as other pediatric etiologies of acute liver failure. In this framework, our group recommends a thorough medical history directed at the age-specific most frequent etiologies (42) and the performance of an abdominal ultrasound with a Doppler evaluation of portal and hepatic veins (in order to rule out the presence of previously undetected structural abnormalities and portal/hepatic thrombosis). Additional subsidiary exams may include screening for metabolic diseases, serologies for other viruses related to hepatitis (hepatitis A, hepatitis B, hepatitis C, cytomegalovirus, Epstein-Bahr virus, etc.), autoantibodies (anti-LKM-1, anti-smooth muscle, anti-nuclear antibodies), ceruloplasmin, and alfa-1 antitrypsin. Guidance from a pediatric hepatologist is highly recommended as a component of the multidisciplinary care of pediatric patients with liver test abnormalities.

In case of a high suspicion of associated and previously unknown hepatic disorders in the newly diagnosed liver damage, and if the diagnostic strategy above is not capable of identifying the possible etiology, abdominal Computed Tomography (CT) and magnetic resonance imaging can be indicated as second-line tests (42). In clinically stable patients, liver biopsies can be indicated after a discussion of the weight of pros and cons with surgeons, intensivists, and hepatologists.

Subsequent evaluation of patients must include periodic repetition of the initial tests, especially when major instabilities occur. Because of the frequent association between PIM-TS and ALT/AST elevations (more frequent in this group of patients), an echocardiogram with evaluation of coronary arteries may be indicated, due to the potential presentation of these patients with coronary artery dilation/aneurysm.

In the context of COVID-19 related coagulopathy, evaluation of liver function based on classical coagulation tests such as prothrombin time and activated partial thromboplastin time (aPTT) can be misleading. In tertiary or quaternary centers, Factor V activity tests (and the Factor V/Factor VIII relation) may be an alternative to better evaluate coagulation factor synthesis in an environment where many potential impediments may hamper the use of traditional coagulation essays.

For patients with previous hepatic conditions and/or cirrhosis, myocardiopathy decompensation can be a concern, sequential evaluation of myocardial enzymes (Troponin and CK-MB) may be suggested. In this group of patients, close monitoring for the development of disease-related complications, such as variceal upper gastrointestinal hemorrhage, spontaneous bacterial peritonitis, and acute or chronic liver failure, must be performed by the clinical team. Liver function tests must be closely monitored, and oscillations from baseline should prompt consultation with pediatric hepatologists.

Management of pediatric COVID-19 patients with liver damage includes the same general considerations as previously published by Carlotti et al. (43). Adequate hemodynamic and ventilatory support is fundamental to minimize the hypoxic and ischemic components of liver damage. It is also important to consider alternatives to potentially hepatotoxic drugs, mainly in patients with ALT/AST elevations and previous hepatic conditions. Given the potential role of microthrombotic events in the pathogenesis of liver damage, the institution of thromboprophylaxis based on solid institutional guidelines must be strongly considered. Empiric antibiotic treatment for bacterial infections is recommended, with coverage to the potential foci in the context of sepsis and bacterial translocation.

Patients with progressively higher ALT/AST levels, associated with ascending direct bilirubin, coagulopathy, and/or hepatic encephalopathy, should be evaluated by a hepatic transplant team. Our institutions’ pediatric liver transplant team advocates the use of King’s College Criteria (44) as a prognostic scoring system to define patients prioritized for liver transplantation in an environment of fulminant/acute liver failure. In the event of a concomitant SARS-CoV-2 infection, liver transplantation should be carefully discussed between the clinical team, the surgical team, and the patients’ family members. The medical team should discuss the risks and benefits of the procedure and the consequent immunosuppressive therapy.

Clinical follow-up after ICU discharge is recommended, with close monitoring of the resolution of liver enzyme elevations and other liver function abnormalities. When unresolved elevated ALT/AST or other alterations are identified, our group strongly suggests consultation with a hepatology specialist in order to rule out other possible causes.

Limitations to the development of the clinical protocol above include a lack of robust clinical evidence as a result of a novel infectious process. Recommendations were made based on observational cohorts, case series, and expert opinions.

This may be the first pediatric COVID-19 protocol focused on the management and follow-up of patients who have developed hepatic damage. As researchers acquire further knowledge on the various physiopathological mechanisms of SARS-CoV-2 infection and the related organ dysfunction, the clinical capacity to provide support and treatment for COVID-19 patients will improve, guided by superior structured clinical protocols.

## AUTHOR CONTRIBUTIONS

Luglio M, Bastos KLM, and Rodrigues IS conceptualized and designed the study, drafted the initial manuscript and reviewed and revised the manuscript. Johnston C and Tannuri U conceptualized and designed the study, supervised data collection, and reviewed and revised the manuscript. Delgado AF and de Carvalho WB conceptualized and designed the study, supervised data collection, and critically reviewed the manuscript for important intellectual content.

All authors approved the final manuscript as submitted and agree to be accountable for all aspects of the work.

## Figures and Tables

**Table 1:** Liver Test Abnormalities (potentially) related to COVID-19 hepatic damage.*

Proposed Mechanism	ALT/AST	GGT/ALP	Bilirubin
Inflammatory Response	Elevated	Variable	Elevated
Drug-induced Liver Injury	Elevated	Variable	Variable
Direct Viral Infection on Cholangiocytes	Variable	Elevated	Elevated
Hypoxic-ischemic/micro-thrombosis	Elevated	Variable	Elevated

* Modified from Morgan et al. (13).

**Table 2:** Main Category A and B Hepatotoxic Drugs Potentially used in Critically ill COVID-19 Pediatric Patients.

Hepatotoxic Drugs	
Category A	Category B
Amoxicillin-Clavulanate	Azithromycin
Carbamazepine	Heparin
Chlorpromazine	Levofloxacin
Paracetamol	Oxacillin
Erythromycin	Phenobarbital
Halothane	
Ibuprofen	
Phenytoin	
Sulfamethoxazole-Trimethoprim	
Valproate	
